# Solving Exact Cover Instances with Molecular-Motor-Powered Network-Based Biocomputation

**DOI:** 10.1021/acsnanoscienceau.2c00013

**Published:** 2022-06-23

**Authors:** Pradheebha Surendiran, Christoph Robert Meinecke, Aseem Salhotra, Georg Heldt, Jingyuan Zhu, Alf Månsson, Stefan Diez, Danny Reuter, Hillel Kugler, Heiner Linke, Till Korten

**Affiliations:** † NanoLund and Solid State Physics, Lund University, Box 118, Lund SE-22100, Sweden; ‡ Center for Microtechnologies, Technische Universität Chemnitz, Chemnitz D-09126, Germany; § Department of Chemistry and Biomedical Sciences, Linnaeus University, Kalmar SE-39231, Sweden; ∥ Fraunhofer Institute for Electronic Nano Systems ENAS, Chemnitz, D-09126, Germany; ⊥ B CUBE - Center for Molecular Bioengineering, Technische Universität Dresden, Dresden D-01307, Germany; # Cluster of Excellence Physics of Life, Technische Universität Dresden, Dresden D-01307, Germany; ∇ Max Planck Institute of Molecular Cell Biology and Genetics, Dresden D-01307, Germany; ○ Faculty of Engineering, Bar-Ilan University, Ramat Gan 5290002, Israel

**Keywords:** parallel computing, computational nanotechnology, molecular motors, biocomputation, nanobiotechnology, biofunctionalization

## Abstract

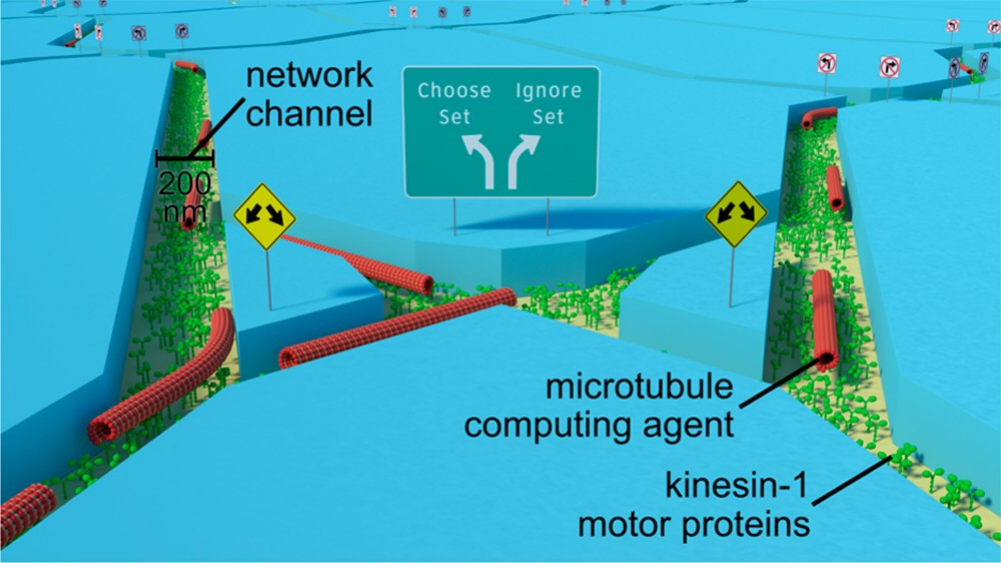

Information processing by traditional, serial electronic processors consumes an ever-increasing part of the global electricity supply. An alternative, highly energy efficient, parallel computing paradigm is network-based biocomputation (NBC). In NBC a given combinatorial problem is encoded into a nanofabricated, modular network. Parallel exploration of the network by a very large number of independent molecular-motor-propelled protein filaments solves the encoded problem. Here we demonstrate a significant scale-up of this technology by solving four instances of Exact Cover, a nondeterministic polynomial time (NP) complete problem with applications in resource scheduling. The difficulty of the largest instances solved here is 128 times greater in comparison to the current state of the art for NBC.

By 2030 the fast-growing information and communication technology sector is expected to consume 20% of the global electricity production.^1^ At the same time, further improvements in energy efficiency of electronic computers are slowed down^2^ by heat generation, small-scale, e.g. quantum, effects, and rising costs.^3^ To help address these challenges, efforts are underway to develop alternative, parallel computing paradigms with the potential to fundamentally reduce the energy consumption of computing.^4^ Such alternative approaches include quantum computation,^5^ biomolecular-motor-based computing,^6^ and network-based biocomputing (NBC).^7^ In NBC, a given combinatorial problem instance is encoded into a graphical, modular network that is embedded in a nanofabricated planar device. This physical network is then explored by a large number of independent cytoskeletal filaments propelled with high energy efficiency by biomolecular motors to find all possible solutions of the given instance.

The main merits of NBC in comparison to electronic computers are that computing units—in the form of cytoskeletal filaments—can work in a highly parallel fashion, are available in large numbers, and have been optimized by evolution to be highly energy efficient. As a result, it has been estimated that NBC uses several orders of magnitude less energy per operation than an electronic computer.^8^ For the purpose of this work, we define the “difficulty” of solving an instance of an NP-complete combinatorial problem by the number of potential solutions that need to be tested to solve this instance. Because of the algorithmic complexity of NP-complete problems, this number grows exponentially with the number of elements in the instance. Note that the difficulty is a property of individual instances, while the algorithmic complexity is a property of the problem as a whole. As a proof of concept of NBC, a very small instance of the NP-complete problem Subset Sum, with a difficulty of eight, has previously been solved.^8^ However, to show that NBC is a viable technology, it is necessary to demonstrate (i) the applicability to other problems of practical importance and (ii) a significant scale-up of the technology. Here we address both challenges by using NBC powered by actin–myosin II^9^ and microtubule–kinesin-1^10^ molecular-motor systems (i) to solve several instances of Exact Cover (ii) that are up to 128 times more difficult than the previous proof of concept Subset Sum instance solved by NBC.^8^ These are important steps that are necessary to understand the potential of the NBC technology.

## Exact Cover Network Algorithm

Exact Cover is a nondeterministic polynomial time (NP) complete problem that has practical applications in resource scheduling, such as airline fleet planning^11^ and allocation of cloud computing resources.^14^ NP-complete problems require the exploration of a solution space that grows exponentially with problem size, and they are difficult to solve using conventional—sequentially operating—electronic computers.^15^ Exact Cover is mathematically defined as follows: given a collection *S* of subsets, each containing elements of a target set *X*, Exact Cover asks whether a subcollection S* of *S* exists, such that each element in *X* is contained in exactly one subset in S*. In other words, the instance has an Exact Cover when all subsets in S* (i) are pairwise disjoint (i.e., ∀*S*_*i*_,*S*_*j*_∈S*. *S*_*i*_∩*S*_*j*_ = ϕ) and (ii) yield *X* when they are joined together (i.e., *S* = ∪_*S*_*i*_∈S*_S_*i*_). For a five-set instance, given the target set *X* = {1,2,3,4,5} and the collection of subsets *S* = [{4,5}, {2,3,5}, {1,4}, {1,3}, {1,2}], the subcollection S* = [{2,3,5}, {1,4}] is a solution (Figure 2A). On the other hand, the subcollection S** = [{4,5}, {2,3,5}] is not a solution because “1” is missing and “5” appears twice. For the purpose of this work, we use the following naming scheme to name specific instances: *E*⟨difficulty⟩_⟨number of solutions⟩_. For example, E16_0_ would be an instance with a difficulty of 16 (four sets in *S*) and no solution. Many sophisticated algorithms have been developed to solve Exact Cover problems. One of these, DLX, uses specific data structures (Dancing links)^16^ and is widely used to address applications such as data clustering.^17^ However, in the worst case, the processing times and energy consumption of these algorithms still increase exponentially with the number of elements in *S*.^16,17^

**Figure 1:**
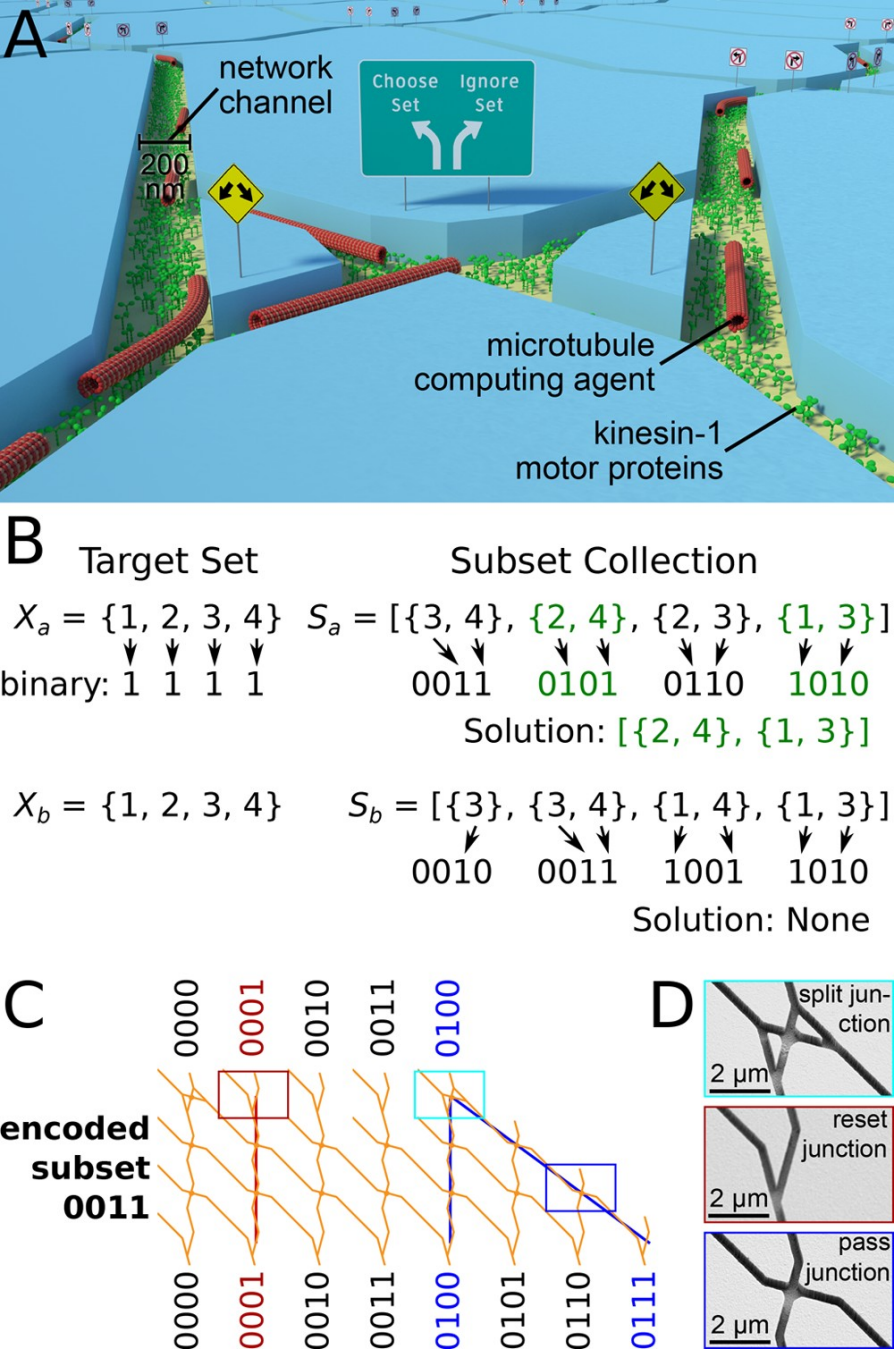
Encoding exact cover into network format. (A) Schematic rendering of a biocomputation network where kinesin-1 motors coat the bottom of 200 nm wide channels etched in poly(methyl methacrylate) (PMMA). The channels are explored by microtubules propelled by kinesin-1 motors. Note that, for the actin–myosin system, the channels were only 100 nm wide. (B) Binary encoding principle: elements of the target set *X* are mapped to bits of a binary number. Here, 1 is mapped to the most significant bit and 4 is mapped to the least significant bit. Each subset in *S* is converted to a binary number by the same mapping rule: i.e., a bit is set to 1 if the respective element in *X* is a member of the subset and set to 0 otherwise. Two examples of exact cover instances (*X*_*a*_*S*_*a*_ and *X*_*b*_*S*_*b*_) are given. *X*_*a*_*S*_*a*_ has a solution (highlighted in green) and *X*_*b*_*S*_*b*_ does not. (C) Example network block that encodes the subset 0011. Agents arriving at input 0100 (blue) encounter a *split junction* (example denoted by a cyan rectangle) that allows the agents to randomly choose (diagonal path) or disregard (the path straight down) the subset encoded by this network block. If the subset is chosen, the number 0011 is added to the input 0100 and the agent arrives at the output 0111. Otherwise, the agent stays in the input column 0100. Because input subsets containing identical elements shall not be combined (as this would violate the rules of exact cover), all inputs that contain elements already present in the encoded subset start with a *reset junction* (example denoted by a red rectangle). This junction forces the agents to take the path straight down: i.e., disregarding the encoded subset. For example, an agent entering at input 0001 (red rectangle) can only leave at output 0001. The input and output rows are separated by two rows of *pass junctions* (example denoted by a blue rectangle) that force agents to continue a diagonal path, incrementing the value of the binary number, or continue moving directly down and maintaining the value of the binary number. Thus, the number of *pass junction* rows ultimately defines which elements are contained in the encoded subset. A full network can contain many network blocks. The output of each network block constitutes the input to the next network block, enabling the combination of many subsets (see Figures 2 and 4 for examples). (D) Scanning electron micrographs of examples for each junction type. The color of the frame corresponds to the rectangles in (C). Note that the SEM images were taken for junctions etched in SiO_2_ instead of PMMA, because PMMA decomposes under electron radiation. Adapted with permission under a Creative Commons Attribution 4.0 license^12^ from ref (13). Copyright 2021 IOP Publishing.

We recently reported a network algorithm for Exact Cover that includes several optimization steps.^13^ The network algorithm enables agents exploring the network to find the solution by randomly choosing all possible subcollections of *S* and checking whether any combination exactly covers *X*. The result for an Exact Cover instance encoded by a particular network is then given by whether agents arrive at the exit that corresponds to the target set *X* (see Figure 1 for a detailed explanation). The scaling behavior of Exact Cover networks with problem size has been discussed in detail by Korten et al.^13^ Briefly, the networks scale approximately linearly with the number of sets in *S* and exponentially with the number of elements in *X.* In the following, we demonstrate the experimental implementation of this algorithm by fabricating four different networks, each encoding an instance of Exact Cover. Two of the instances had 5 sets and were solved using actin filaments as computing agents and powered by myosin II as motor proteins. The other two instances had 10 sets and were solved using microtubules as computing agents and powered by kinesin-1 as motor proteins. A recent review details the advantages and disadvantages of using the actin–myosin vs the microtubule–kinesin-1 systems for nanotechnological applications.^18^ Briefly, actin is smaller, faster, and more flexible, enabling faster calculations and smaller network dimensions, while microtubules are stiffer, reducing error rates.

## Successfully Solving Two Five-Set Exact Cover Instances Using NBC Powered by the Actin–Myosin System

**Figure 2:**
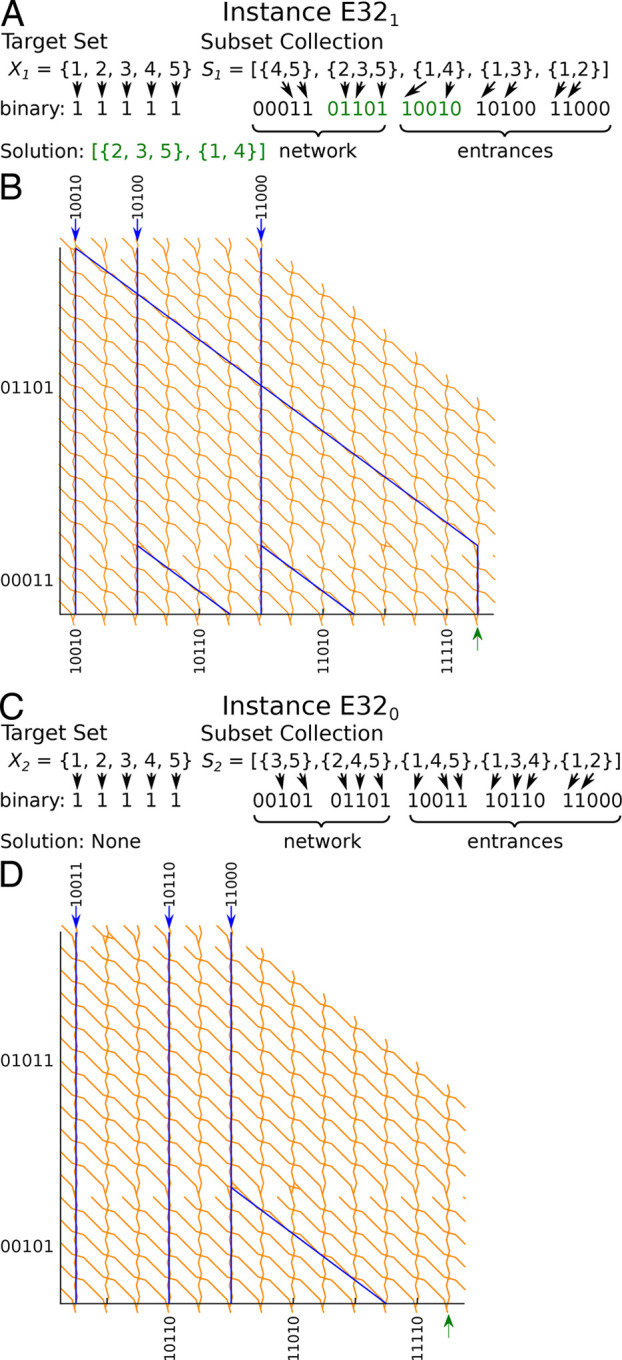
Five-set Exact Cover instances encoded into network format. (A, C) Instance E32_1_ (A) and instance E32_0_ (B) including the binary mapping of the given target sets *X*_1_ and *X*_2_ as well as the subsets in *S*_1_ and *S*_2_ as described in detail in Figure 1B. (B, D) Networks encoding instance E32_1_ (B) and instance E32_0_ (D), which enable agents exploring the respective network to randomly choose subcollections from *S*_1_ or *S*_2_, following the rules defined by Exact Cover as explained in Figure 1C. The solution to the Exact Cover instance encoded by the network can be identified by checking whether agents exit at the binary number that represents the target set (green arrows in (B) and (D)). If a significant number of agents arrive at that particular exit, then the Exact Cover instance does have a solution; otherwise, it does not. (A, B) Exact Cover instance that has a solution. (C, D) Exact Cover instance that has no solution. (B, D) Blue arrows indicate network entrances; correct paths are highlighted in blue. Note that the closed channels at the right edge of the network ensure that filaments reaching these paths are forced to detach from the network and that no correct path leads to these channels. Therefore, only filaments making errors can reach them.

Using the algorithm described above, we encoded two five-set Exact Cover instances into network format: (i) instance E32_1_, *X*_1_ = {1,2,3,4,5}, *S*_1_ = [{4,5}, {2,3,5}, {1,4}, {1,3}, {1,2}] with exactly one solution, [{1,4}, {2,3,5}] (Figure 2A,B); (ii) instance E32_0_, *X*_2_ = {1,2,3,4,5}, *S*_2_ = [{3,5}, {2,4,5}, {1,4,5}, {1,3,4}, {1,2}] with no solution (Figure 2C,D). The networks were fabricated using electron-beam lithography and explored by fluorescently labeled actin filaments that were propelled by myosin II motor fragments and recorded by video fluorescence microscopy.

**Figure 3:**
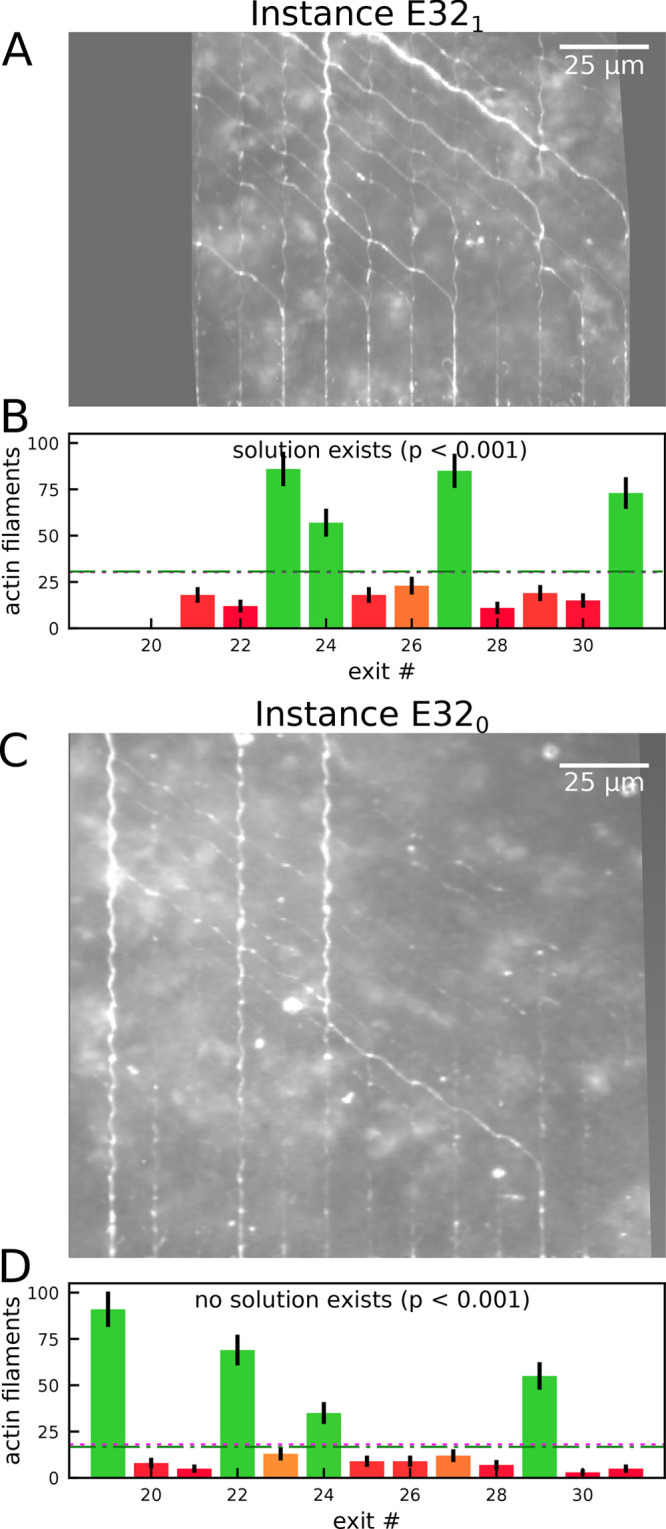
Successful operation of optimized Exact Cover networks with 5 sets (32 potential solutions) by the actin–myosin system. Optimized networks encoding Exact Cover instances E32_1_ (A, B) and E32_0_ (C, D). (A, C) Standard deviation projection of 2500 frames of 0.2 s time-lapse fluorescence micrographs of computing networks. Network paths that were more frequently visited by actin filaments are brighter. The micrograph in (A) has a smaller field of view, because it was acquired with a higher magnification objective. (B, D) Number of actin filaments counted at each exit. RGB colors indicate probabilities that the respective counts correspond to a correct (more green) or an incorrect (more red) exit. Error bars represent the counting error (square root of the respective value). Values above the green dash-dotted lines are significantly correct exits (*p* < 0.05), and values below the magenta dotted lines are significantly incorrect exits (*p* < 0.05). The rightmost exits represent the solution to the Exact Cover instances. The insets give the probabilities that the respective Exact Cover instance had a solution. Probabilities were estimated as described in section S1 in the Supporting Information. In total, the calculations took 0.14 h for each network.

The most frequently traveled paths in the networks (Figure 3A,C) indicated possible solutions to the Exact Cover instances E32_1_ and E32_0_. To decide whether an instance has a solution, we need to check whether a significant number of filaments arrives at the predefined target exit, in this case exit number 31 for both E32_1_ and E32_0_. Indeed, for E32_1_, which has a solution, significantly more filaments arrive at exit 31 (rightmost bar in Figure 3B) in comparison to that for E32_0_ (Figure 3D).

We now need to determine whether these results are statistically significant with respect to the expected background noise (see section S1 in the Supporting Information for details). We do this by first evaluating the error rates within the network. Specifically, by visually evaluating pass junction crossing events, we determined the average ratio of actin filaments taking a turn instead of passing through on a straight path (the *pass-junction* error) to be 3.8% ± 0.4% (*N* = 2349) for E32_1_ and 2.6% ± 0.2% (*N* = 5079) for E32_0_, respectively.

The resolution of our optical microscopes was not sufficient to determine the exact cause of pass junction errors, but we noticed that filaments often got stuck and curled up briefly, before taking a wrong turn. This hints that pass junction errors were caused at least in part by filaments getting stuck at the walls or on motor proteins, curling up, and then being released in the wrong direction. Note that detaching filaments did not contribute to the error rates, because they did not arrive at an exit and thus were not counted. From the pass junction error rates, and from the total number of filaments exiting the network, we estimated the background number of filaments expected to exit at an incorrect position for each network.

Counting the number of filaments arriving at each network exit (Figure 3B,D) showed the expected performance. In particular, the target exits number 31 (rightmost bars in Figure 3B,D) returned the correct results: for E32_1_, the number of filaments arriving at the target exit (rightmost green bar in Figure 3B) was significantly larger than the expected threshold for background noise (green dash-dotted line in Figure 3B). In contrast, the number of filaments arriving at the target exit of E32_0_ (rightmost red bar in Figure 3D) was significantly lower than expected for background noise (magenta dotted line in Figure 3D).

To quantitatively estimate the reliability of our results, we developed a statistical algorithm (see section S1 in the Supporting Information for details). Briefly, this algorithm estimates (i) the fraction of filament paths affected by errors, due to either pass-junction errors or landing errors, (ii) the worst-case number of filaments per exit that have been affected by errors, (iii) the worst-case number of filaments expected to appear at a correct exit, and (iv) the *p* values for the hypotheses that the observed number of filaments corresponds to a correct exit or an incorrect exit, respectively. With this algorithm, we can determine the statistical significance with which the observed filament counts at the network exits correctly indicates whether the respective Exact Cover instance has a solution. In order to get the actual subcollection S* representing the solution, we have two possibilities. (i) We observe the path that the filaments take from an entrance to the target exit, which is easy for networks fitting into the field of view of a microscope (Figure 3B) but can become difficult if the network is larger than the field of view (Figure 3A). (ii) We perform the experiments in several networks, each time removing one of the sets from the networks and checking whether there still is a solution. If there still is a solution, then the removed set was not part of the solution; otherwise, it was.

## Network Encoding of “Reverse” Exact Cover Devices

**Figure 4:**
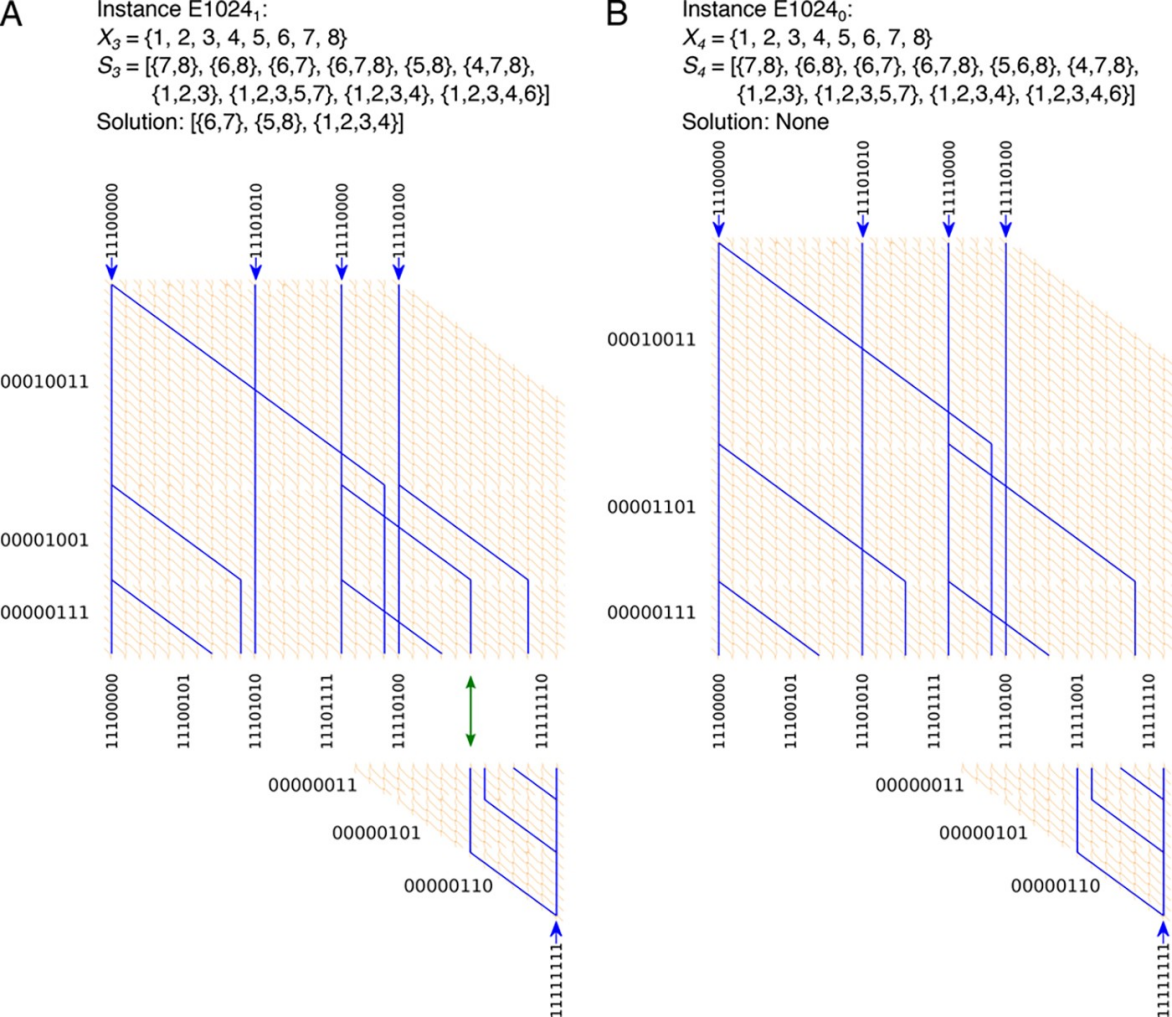
Layouts of reverse Exact Cover devices to solve an Exact Cover problem with 10 sets, which has 1024 solutions. (A) Network layout of the Exact Cover instance E1024_1_ corresponding to the binary numbers 00000011, 00000101 and 00000110, 00000111, 00001001, 00010011, 11100000, 11101010, 11110000, 11110100. This Exact Cover instance has a solution, which is indicated by the green double arrow. (B) Network layout of the Exact Cover instance E1024_0_ corresponding to the binary numbers 00000011, 00000101 and 00000110, 00000111, 00001101, 00010011, 11100000, 11101010, 11110000, 11110100. This Exact Cover instance has no solution. Correct paths are highlighted in blue.

For the larger instances with 10 sets, we used a network encoding that employs what we call “reverse exploration” optimization.^13^ Since we know the target exit, we can split the network up into a top and a bottom part, each encoding only half the total number of sets represented in the network (Figure 4). That way, the network is explored both from the entrances and in reverse from the exit, reducing the size of the top and bottom network parts and thus reducing the number of filaments as well as the time needed to find the solution. We encoded two 10-set Exact Cover instances into network format: E1024_1_, *X*_3_ = {1,2,3,4,5,6,7,8}, *S*_3_ = [{6,7,8}, {5,8}, {4,7,8}, {1,2,3}, {1,2,3,5,7}, {1,2,3,4}, {1,2,3,4,6}, {7,8}, {6,8}, {6,7}] has exactly one solution, [{1,2,3,4}, {5,8}, {6,7}] (Figure 4A); E1024_0_, *X*_4_ = {1,2,3,4,5,6,7,8}, *S*_4_ = [{6,7,8}, {5,6,8}, {4,7,8}, {1,2,3}, {1,2,3,5,7}, {1,2,3,4}, {1,2,3,4,6}, {7,8}, {6,8}, {6,7}] does not have a solution (Figure 4B).

We successfully solved 2 10-set Exact Cover instances using NBC powered by the microtubule–kinesin-1 molecular motor system. The networks were fabricated using electron-beam lithography and explored by fluorescently labeled microtubules that were propelled by kinesin-1 motor proteins^19^ and recorded by 5 s time-lapse fluorescence microscopy. The pass-junction error was 0.03 ± 0.015% *N* = 13263). The reduced pass-junction error allowed microtubules to traverse the networks with more than 34 pass junctions without error with an overall success probability of 98.9% ((1 – 0.0003)^34^). However, still 517 out of a total of 1554 filaments exiting the network made at least one error. The biggest contribution to the error (499 out of 517 filaments that made at least one error) came from microtubules landing from solution at random positions in the network—causing these microtubules to exit at random positions (termed landing error). Interestingly, we did not observe any landing error for actin filaments, despite the fact that we also had filaments in solution above the networks. Actin probably does not land in channels, because several nonprocessive myosin II motors are needed to propel a filament^20^—unlike the processive kinesin-1 where a single motor suffices to pull a microtubule into the channel.^21^

**Figure 5:**
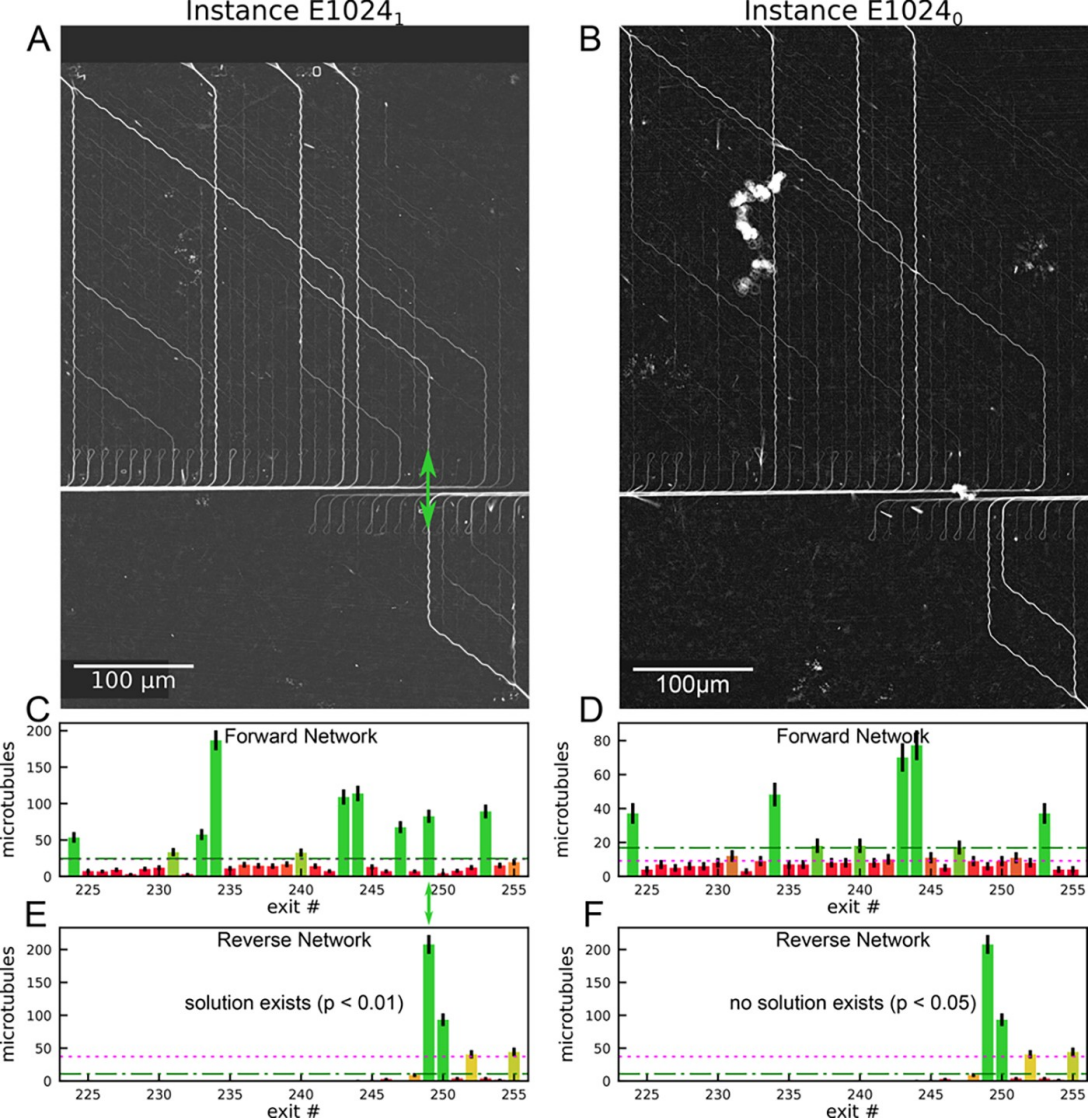
Microtubule results for the large network of Exact Cover instances with 10 sets: (A, C, E) Instance E1024_1_ with exactly one solution (indicated by the green double arrow in (A)). (B, D, F) Instance E1024_0_. (A, B) Standard deviation plots of 3600 frames of 5 s time-lapse fluorescence micrographs of microtubules moving through the computational network. Paths that were more frequently visited by microtubules are brighter. (A) The green double arrow highlights where paths from the forward and reverse network meet, indicating that this instance has a solution. (C–F) Number of microtubules counted at each exit of the (C, D) forward and (E, F) reverse halves of the Exact Cover network. RGB colors indicate probabilities that the respective counts correspond to a correct (more green) or an incorrect (more red) exit. Error bars represent the counting error (square root of the respective value). Values above the green dash-dotted lines are significant correct exits (*p* < 0.05), and values below the magenta dotted lines are significant incorrect exits (*p* < 0.05). In total, the calculation took 6 h for each network.

Despite the observed errors, the most frequently traveled paths in the network (Figure 5A,B) matched the expected paths (blue lines in Figure 4) very well, confirming that the majority of microtubules explored the network as expected. Counting the number of microtubules arriving at each exits confirmed this. The statistical significance of each exit was determined as described in section S1 in the Supporting Information. Values above the green dash-dotted lines are significant correct exits (*p* < 0.05) and are shown in green (in Figure 5C–F). Values below the magenta dotted lines are significant incorrect exits (*p* < 0.05) and are shown in red (in Figure 5C–F).

According to the network algorithm,^13^ the Exact Cover instance has a solution if microtubules exit at corresponding exits in both the forward *and* the reverse networks (see section S1 in the Supporting Information for how a correct solution is determined). For E1024_1_ (Figure 5A,C,E) forward and reverse paths meet at exit 11111001 (green double arrows in Figures 4A and 5A), correctly indicating that the respective Exact Cover instance has a solution. The solution is represented by a complete path from the set {1,2,3,4}—corresponding to entrance (11110000)—via the sets {5,8} (00001001) in the forward network and {6,7} (00000110) in the reverse network to the target set {1,2,3,4,5,6,7,8} (11111111). For E1024_0_ there are no matching exits (Figure 5B,D,F), correctly indicating that no solution exists for the respective Exact Cover instance.

In summary, we successfully employed NBC to experimentally solve 4 instances of Exact Cover, 2 instances with 5 sets and 2 instances with 10 sets, corresponding to difficulties of 32 and 1024 potential solutions, respectively. In comparison to the state of the art for NBC—solving an NP-complete problem with a difficulty of eight potential solutions^8^—our results constitute scale-ups by factors of 4 and 128, respectively. Thus, we have demonstrated a significant scale-up of the NBC technology. This scale-up of NBC was enabled by two factors. (i) Improvements in the network algorithm^13^ allowed us to encode instances with a 128 times greater difficulty into networks with only approximately 13 times more junctions and 4 times more exits in comparison to the state of the art network algorithm for Subset Sum.^8^ (ii) There was a 10 times reduction of the error rates at pass junctions for microtubules in comparison to the state of the art.^8^ This reduction in error rate was achieved by switching to a different channel wall material (PMMA). While the difficulty of the Exact Cover instances we solved here is still small in comparison to what can be solved by an electronic computer^22^ or a quantum annealer,^23^ our results demonstrate not only theoretically^13^ but also experimentally that NBC is applicable to the Exact Cover problem as a whole—another problem of practical importance in addition to Subset Sum.^8^ Moreover, despite the fact that NBC works stochastically, it is possible to achieve results with a confidence similar to that of electronic computers by using a sufficient number of computing agents (see section S2 in the Supporting Information for details).

In conclusion, our results show that NBC (i) can be scaled-up significantly and (ii) is applicable to different combinatorial problems. As a next step, it will be interesting to measure the energy consumed by NBC networks and benchmark their energy consumption against electronic computers. However, this will likely require further scale-up or long-term measurements so that the NBC devices consume measurable amounts of ATP. Because of the different sources of errors, the future scale-up of microtubule- and actin-powered NBC will require different optimizations. (i) Microtubules will require eliminating landing errors—for example, by microfluidic focusing of the filaments to the loading zones of the NBC networks, avoiding the presence of microtubules above the network channels. (ii) Actin filaments will require the redesign of pass junctions in order to reduce the junction errors. This can be realized by narrower channels^8^ or the integration of 3D pass junction geometries with bridges and tunnels^24,25^ that would eliminate pass-junction errors altogether. Despite our significant progress, it is obvious that NBC still has a long way to go if it is to compete with electronic computers that have had a headstart of decades’ (and billions of dollars) worth of research and development. We have recently summarized the requirements we deem necessary for NBC to become competitive.^26^ Briefly, (i) agents need to be supplied to the network in sufficiently large quantities, either through multiplication within the network^27^ or through a sufficient number of entrances, (ii) the physical network needs to be scalable, i.e. more efficient algorithms that enable a reduction in search space similar to the algorithms available for electronic computers are needed,^28^ (iii) methods to store information on the agents would enable much more compact networks that can solve many different instances (unlike the networks shown here that are instance-specific), and (iv) methods to detect single agents and their tags in parallel are needed. Achieving these requirements would enable leveraging the energy efficiency of the molecular motors powering NBC: we estimate the energy consumption of our networks to be ∼4 × 10^–15^ J/operation, orders of magnitude less than the (2–6) × 10^–10^ J/operation of an electronic computer^8^ (see section S3 in the Supporting Information for details).

## Supporting Information Available

Supplementary methods, number of filaments required for a target confidence level of the computation, and estimated energy consumption per operation (PDF)

## References

[ref1] Andrae A. S. G.; Edler T. On Global Electricity Usage of Communication Technology: Trends to 2030. Challenges 2015, 6 (1), 117–157. 10.3390/challe6010117.

[ref2] Sun Y.; Agostini N. B.; Dong S.; Kaeli D. Summarizing CPU and GPU Design Trends with Product Data. arXiv:1911.11313 [cs] 2020.

[ref3] Arden W.; Brillouët M.; Cogez P.; Graef M.; Huizing B.; Mahnkopf R. More-than-Moore” White Paper. http://www.itrs2.net/uploads/4/9/7/7/49775221/irc-itrs-mtm-v2_3.pdf (accessed 2022-02-22).

[ref4] Konopik M.; Korten T.; Lutz E.; Linke H. Fundamental Energy Cost of Finite-Time Computing. arXiv:2101.07075 [cond-mat] 2021. 10.1038/s41467-023-36020-2 PMC9883481 36707510

[ref5] Bengtsson A.; Vikstål P.; Warren C.; Svensson M.; Gu X.; Kockum A. F.; Krantz P.; Križan C.; Shiri D.; Svensson I.-M.; Tancredi G.; Johansson G.; Delsing P.; Ferrini G.; Bylander J. Improved Success Probability with Greater Circuit Depth for the Quantum Approximate Optimization Algorithm. Phys. Rev. Applied 2020, 14 (3), 034010 10.1103/PhysRevApplied.14.034010.

[ref6] Kabir A. Md. R.; Kakugo A. Biomolecular Motor-Based Computing. In Handbook of Unconventional Computing; World Scientific: 2021; WSPC Book Series in Unconventional Computing, pp 451–464. 10.1142/9789811235740_0015.

[ref7] Nicolau D. V.; Nicolau Jr D. V.; Solana G.; Hanson K. L.; Filipponi L.; Wang L.; Lee A. P. Molecular Motors-Based Micro- and Nano-Biocomputation Devices. Microelectron. Eng. 2006, 83 (4–9), 1582–1588. 10.1016/j.mee.2006.01.198.

[ref8] Nicolau D. V.; Lard M.; Korten T.; van Delft F. C. M. J. M.; Persson M.; Bengtsson E.; Mansson A.; Diez S.; Linke H.; Nicolau D. V. Parallel Computation with Molecular-Motor-Propelled Agents in Nanofabricated Networks. Proc. Natl. Acad. Sci. U.S.A. 2016, 113 (10), 2591–2596. 10.1073/pnas.1510825113. 26903637 PMC4791004

[ref9] Iino R.; Kinbara K.; Bryant Z. Introduction: Molecular Motors. Chem. Rev. 2020, 120 (1), 1–4. 10.1021/acs.chemrev.9b00819. 31910626

[ref10] Tsitkov S.; Hess H. Design of Active Nanosystems Incorporating Biomolecular Motors. In Out-of-Equilibrium (Supra)molecular Systems and Materials; Wiley: 2021; pp 379–422. 10.1002/9783527821990.ch13.

[ref11] Vikstål P.; Grönkvist M.; Svensson M.; Andersson M.; Johansson G.; Ferrini G. Applying the Quantum Approximate Optimization Algorithm to the Tail-Assignment Problem. Phys. Rev. Applied 2020, 14 (3), 034009 10.1103/PhysRevApplied.14.034009.

[ref12] Creative Commons-Attribution 4.0 International-CC BY 4.0. https://creativecommons.org/licenses/by/4.0/ (accessed 2022-05-27).

[ref13] Korten T.; Diez S.; Linke H.; Nicolau D. V.; Kugler H. Design of Network-Based Biocomputation Circuits for the Exact Cover Problem. New J. Phys. 2021, 23 (8), 085004 10.1088/1367-2630/ac175d.

[ref14] Ba C. An Exact Cover-Based Approach for Service Composition. 2016 IEEE International Conference on Web Services (ICWS) 2016, 631–636. 10.1109/ICWS.2016.87.

[ref15] Garey M. R.; Johnson D. S. Computers and Intractability: A Guide to the Theory of NP-Completeness, 1st ed..; W. H. Freeman: 1979.

[ref16] Knuth D. E. Dancing Links. arXiv:cs/0011047 [cs] 2000.

[ref17] Chabert M.; Solnon C. A Global Constraint for the Exact Cover Problem: Application to Conceptual Clustering. Journal of Artificial Intelligence Research 2020, 67, 509–547. 10.1613/jair.1.11870.

[ref18] Reuther C.; Catalano R.; Salhotra A.; Vemula V.; Korten T.; Diez S.; Månsson A. Comparison of Actin- and Microtubule-Based Motility Systems for Application in Functional Nanodevices. New J. Phys. 2021, 23 (7), 075007 10.1088/1367-2630/ac10ce.

[ref19] Howard J.; Hudspeth A. J.; Vale R. D. Movement of Microtubules by Single Kinesin Molecules. Nature 1989, 342 (6246), 154–158. 10.1038/342154a0. 2530455

[ref20] Rastogi K.; Puliyakodan M. S.; Pandey V.; Nath S.; Elangovan R. Maximum Limit to the Number of Myosin II Motors Participating in Processive Sliding of Actin. Sci. Rep 2016, 6 (1), 32043 10.1038/srep32043. 27554800 PMC4995457

[ref21] Hancock W. O.; Howard J. Kinesin’s Processivity Results from Mechanical and Chemical Coordination between the ATP Hydrolysis Cycles of the Two Motor Domains. Proc. Natl. Acad. Sci. U.S.A. 1999, 96 (23), 13147–13152. 10.1073/pnas.96.23.13147. 10557288 PMC23915

[ref22] Colbourn C. J.; Dinitz J. H. The CRC Handbook of Combinatorial Designs; CRC Press: 2007; Vol. 5005.

[ref23] Willsch D.; Willsch M.; Gonzalez Calaza C. D.; Jin F.; De Raedt H.; Svensson M.; Michielsen K. Benchmarking Advantage and D-Wave 2000Q Quantum Annealers with Exact Cover Problems. Quantum Inf Process 2022, 21 (4), 141 10.1007/s11128-022-03476-y.

[ref24] Lard M.; ten Siethoff L.; Generosi J.; Månsson A.; Linke H. Molecular Motor Transport through Hollow Nanowires. Nano Lett. 2014, 14 (6), 3041–3046. 10.1021/nl404714b. 24874101

[ref25] Reuther C.; Steenhusen S.; Meinecke C. R.; Surendiran P.; Salhotra A.; Lindberg F. W.; Månsson A.; Linke H.; Diez S. Molecular Motor-Driven Filament Transport across Three-Dimensional, Polymeric Micro-Junctions. New J. Phys. 2021, 23 (12), 125002 10.1088/1367-2630/ac39b4.

[ref26] Zhu J.; Korten T.; Kugler H.; van Delft F.; Mansson A.; Reuter D.; Diez S.; Linke H. Physical Requirements for Scaling up Network-Based Biocomputation. New J. Phys. 2021, 23 (10), 105004 10.1088/1367-2630/ac2a5d.

[ref27] Reuther C.; Santos-Otte P.; Grover R.; Heldt G.; Woehlke G.; Diez S. Multiplication of Motor-Driven Microtubules for Nanotechnological Applications. Nano Lett. 2022, 22 (3), 926–934. 10.1021/acs.nanolett.1c03619. 35050639

[ref28] Junttila T.; Kaski P. Exact Cover via Satisfiability: An Empirical Study. In Principles and Practice of Constraint Programming - CP 2010; Cohen D., Ed.; Springer: 2010; Lecture Notes in Computer Science, pp 297–304. 10.1007/978-3-642-15396-9_25.

