# Supplementary material for: Solving Exact Cover Instances with Molecular-Motor-Powered Network-Based Biocomputation
Source: ACS Nanosci Au. 2022 Jun 23;2(5):396–403. doi: 10.1021/acsnanoscienceau.2c00013 (PMC9585575; doi:10.1021/acsnanoscienceau.2c00013)
Supplement: Supplementary file 1 — ng2c00013_si_001.pdf [file ng2c00013_si_001.pdf]

Solving Exact Cover Instances with Molecular-Motor-Powered Network-Based Biocomputation

Supplementary Material

Pradheebha Surendiran[1]‡, Christoph Robert Meinecke[2]‡, Aseem Salhotra[3], Georg Heldt[4], Jingyuan Zhu[1], Alf Månsson[3], Stefan Diez[5,6,7], Danny Reuter[2,4], Hillel Kugler[8], Heiner Linke[1], Till Korten*[5]

1. NanoLund and Solid State Physics, Lund University, Lund, SE-22100, Sweden
2. Center for Microtechnologies, Technische Universität Chemnitz, Chemnitz, D-09126, Germany
3. Department of Chemistry and Biomedical Sciences, Linnaeus University, Kalmar, SE-39231 Sweden
4. Fraunhofer Institute for Electronic Nano Systems ENAS, Chemnitz, D-09126, Germany
5. B CUBE - Center for Molecular Bioengineering, Technische Universität Dresden, D-01307, Germany
6. Cluster of Excellence Physics of Life, Technische Universität Dresden, D-01307, Germany
7. Max Planck Institute of Molecular Cell Biology and Genetics, Dresden, D-01307, Germany
8. Faculty of Engineering, Bar-Ilan University, Ramat Gan, IL 5290002, Israel

*Corresponding author: till.korten@tu-dresden.de ‡These authors contributed equally

S1 Supplementary methods

Fabrication of actin-myosin-system networks. NBC-devices for the actin-myosin-system were fabricated using 10 × 8 mm^2 Si (100) substrates. A 75 nm thick SiO_2 layer on the Si-substrate was made by thermal oxidation. The substrates were ashed in oxygen plasma at 5 mbar for 60 s and cleaned in acetone and isopropanol for 3 min each in an ultrasonic bath at room temperature followed by drying under nitrogen flow. CSAR 62 (Allresist GmbH, Strausberg, Germany) was spin-coated to a thickness of approximately 400 nm and baked on a hotplate at 180°C for 120 s. The network consisting of 100nm-wide channels was patterned by electron beam lithography (Raith Voyager) with a dose of 150 µC/cm^2. The CSAR 62 chips were immersed in the developer amyl acetate for 90 s and rinsed in IPA for 30 s, followed by drying under nitrogen flow. After development, the chips were oxygen plasma ashed at 5 mbar for 45 s which would make the CSAR 62 hydrophilic thereby not supporting the actin attachment and giving selective guiding between the exposed channel floors and the resist walls[1,2]. The samples were then silanized with trimethylchlorosilane in a controlled chamber at 200 mbar to realize desired surface chemistry for supporting actin motility on exposed SiO_2 surfaces.

Fabrication for a glass-based bio-functionalization of the microtubule-kinesin-1-system. The NBC used for these experiments was fabricated on 100 mm glass wafers of 200 µm thickness. After an O_2 plasma cleaning and surface activation step for 30 seconds, a resist layer poly(methyl methacrylate) (PMMA) (ALLRESIST AR-P 679.03) was spin-coated onto the substrate, to a thickness around 400 nm and hard-baked at 180°C for 5 minutes. Afterward an additional electrically conductive resist layer Electra 92 (ALLRESIST AR-PC 5090.02) was spin-coated and soft baked at 90°C for 2 minutes. The 200 nm-wide channels were patterned by electron beam lithography (Vistec SB254, Vistec GmbH) at 5 kV using a dose of 500 µC/cm^2 and subsequently flushed with deionized water to remove the exposed Electra resist. The PMMA was developed for 60 s in a mixture of methyl isobutyl ketone and isopropanol (1:3), rinsed in isopropanol followed by deionized water, and dried in an oven. After preparation of devices of around (10 mm)^2 size.

Actin-myosin gliding assays were performed as previously described[3]. Briefly, flow cells were built by gluing the device face down to microscope coverslips (No. 0, Menzel-Gläser, Braunschweig, Germany) using two stretches of parafilm. The *in-vitro* motility assays were performed at 25°C: 120 µg/ml heavy meromyosin was incubated for 5 min, followed by 1 mg/ml bovine serum albumin (2 min), and by rinsing with wash buffer. The flow cell was then incubated with blocking actin (non-fluorescent actin filaments dissolved in wash buffer; 1 µM). Next, the flow cell was incubated with Magnesium Adenosine Triphosphate (MgATP in wash buffer; 1 mM) for 2 min, followed by two volumes rinsing with wash buffer, and then further incubation with rhodamine-phalloidin labelled actin filaments (in wash buffer; 10-15 nM), followed by one volume rinsing with wash buffer. Finally, assay buffer was added followed by incubation for 2 min before imaging using the microscope.

Microtubule-kinesin-1 gliding assay. To achieve optimal guiding[4,5], we used a combination of physical guiding walls and chemical passivation, adapted from

previous protocols[6–8]. Briefly, the PMMA surface of the computational chip was passivated by incubating for 2 h with 1% w/v F127 (Sigma-Aldrich) in BRB80 (80 mM PIPES/KOH, pH 6.9, 1mM EGTA, 1mM $MgCl_2$) at room temperature to prevent protein binding anywhere except on the glass bottom of the channels. Flow cells were constructed by placing stretched stripes of parafilm on the chips next to the structures. The channels were closed with a glass coverslip (Menzel, (18×18) mm^2) silanized with 2-[Methoxy(poly-ethyleneoxy) propyl] trimethoxy-silane] (PEG-silane) 90% (ABCR, SIM4492.7; 0.23% v/v in toluene-HCl) overnight at room temperature . Flow cells were perfused with casein-containing solution (0.5 mg ml−1) in BRB80 and left to adsorb for 5 min. Next, 5 µl of 4 nM kinesin-1 solution (was perfused into the flow cells and incubated for another 10 min. Thereafter, a motility solution (1 mM MgATP, 20 mM D-glucose, 20 µg/ml glucose oxidase, 10 µg/ml catalase, 10 mM DTT, 10 µM taxol in BRB80) containing rhodamine-labeled, taxol-stabilized microtubules was applied. Full-length kinesin-1 from Drosophila was expressed in insect cells and purified as described previously[8]. Tubulin was isolated from the porcine brain and subsequently labeled with rhodamine as described previously[9].

Image processing actin-myosin-system. The motility assays were imaged using a Zeiss inverted microscope with a Mercury lamp, 63x (1.4 NA) and 40x (1.3 NA) oil immersion objectives and a rhodamine filter set (excitation: 545/20 nm, dichroic: 562, emission: 591/43 nm,). The recordings were captured at a framerate of 5 frames per second using an EMCCD camera (Hamamatsu C9100) and analyzed with ImageJ[10].

Image processing microtubule-kinesin-1-system. Fluorescence time-lapse movies were recorded using an Eclipse Ni upright optical microscope (Nikon) with a rhodamine filter set (AHF; excitation: 550/20, dichroic: 562, emission 607/70). Time-lapse images were recorded at a rate of 0.2 frames per second with an exposure time of 100 ms using a CMOS camera (ORCA-fusion C14440, Hamamatsu) in conjunction with NIS Elements imaging software (Nikon).

Statistical data evaluation. We estimated the probability that the results measured for each network indicate that the corresponding problem instance has exact cover in four steps:

i) Estimate the number of filaments that have been affected by errors. We measured junction error rates by observing filaments crossing pass junctions. Landing errors were estimated by comparing experimental results with Monte Carlo simulations. With these two errors, the number of filaments that have been affected by errors was estimated as follows:

$$e_a = \frac{n_{tot} - n_{tot}(1-e_p)^{r_p} + l*j}{x} \quad (1)$$

Where e_a: average number of filaments affected by errors; n_{tot}: total number of filaments exiting the network; e_p: pass-junction error; r_p: number of pass junction rows; l: landing error per junction; j: number of junctions in the network. x: number of exits.

ii) Estimate the worst-case number of filaments per exit that have been affected by errors. The error estimation described above assumes an even distribution of errors for all junctions. However, this is not the case, because not all junctions are split junctions in a split/pass junction row. Therefore, the error estimation described above was performed separately for each set encoded in the network (for each block of junctions from one split/pass junction row to the next). Thus, the worst-case error was estimated by the following algorithm:

$$e_w = 0.5\, e_{prev} + e_{cur} \quad (2)$$

Where e_w: worst-case error; e_{prev}: average error of the block of junctions encoding the previous set; e_{cur}: average error estimated for the block of junctions encoding the current set using equation (1). Eq. (2) was applied successively for each junction block in the respective network from top to bottom. The overall worst-case error was the worst-case error of the last junction block.

iii) Estimate the worst-case average number of filaments expected to appear at a correct exit. We can estimate the total number of filaments without error from equation (1) as:

$$n_c = n_{tot} - e_a * x \quad (3)$$

We also know the total number of possible paths through the network:

$$p = E * 2^{s_n} \quad (4)$$

From equations (3) and (4) we get the average expected number of filaments per path that does not contain any reset junctions. Added to the average error e_a from Eq. (1), this corresponds to the worst-case average number of filaments expected to appear at a correct exit:

$$n_{min} = \frac{n_c}{p} + e_a \quad (5)$$

iv) Estimate the p-values for the hypotheses that the observed number of filaments corresponds to a correct exit or an incorrect exit, respectively. We assumed that the number of filaments observed at each exit (n) is a sample from a normal distribution with a mean and standard deviation equal to n and $\sqrt{n}$,

respectively. Based on this assumption, we used a cumulative distribution function to estimate the probability for the null hypothesis of a correct exit (i.e. that the number of filaments observed is less than or equal to the worst-case error):

$$p_{cor} = \text{cdf}(n < e_w) \qquad (6)$$

and for the null hypothesis of an incorrect exit (i.e. that number of filaments observed is greater than or equal to the expected number of filaments per path):

$$p_{err} = \text{cdf}(n > n_{min}) \qquad (7)$$

For the single network encoding used with actin filaments, the result was verified by comparing p_{cor} and p_{err} to the designated significance level of 0.05.

For the reverse network encoding (used with microtubules), we first used equations (6) and (7) to determine whether individual exits of the forward- (p_i^f) and reverse (p_i^r) networks were significantly correct (p_i^{fc} / p_i^{rc}) or significantly incorrect (p_i^{fe} / p_i^{re}). The overall answer was then determined by the following Boolean formula for whether a solution exists:

$$\exists(p_i^{fc} < 0.05 \ \wedge \ p_i^{rc} < 0.05) \qquad (8)$$

and for whether no solution exists:

$$\forall(p_i^{fe} < 0.05 \vee p_i^{re} < 0.05) \qquad (9)$$

S2 Number of filaments required for a target confidence level of the computation

We first estimate the necessary number of agents needed to solve the combinatorial problem with sufficiently strong confidence.

Estimating the confidence level for actin filaments

In NBC, exploration of all possible paths of the network by the agents effectively solves the interested problem. Konopik et al, [11] estimated the number of NBC agents needed to solve a given combinatorial problem with a given confidence level α. Briefly, assuming ideal split junctions (with a split ratio of 50%) and ideal pass junctions (the agents keep on moving along the vertical/diagonal path), we get:

$$N_{ideal} = \frac{l}{2p_i}\left(l * q_i + \frac{2}{l}n_i + \sqrt{l^2 * q_i^2 + 4q_i n_i}\right)$$

In which p_i is the probability of filaments at exit i in the network; $q_i = 1 - p_i$; n_i is the number of agents in exit i; l is the numerical measurement that describes a value's relationship to the mean of the group of values, it is calculated for a desired confidence level α through the cumulative distribution function of the normal distribution. This ideal case was then extended to non-ideal networks with a given pass junction error p_{PJ}:

$$N_{non-ideal} = \frac{l}{2p_{PJ}^c}\left(l * q_{PJ}^c + \frac{2}{l}N_{ideal} + \sqrt{l^2 * {q_{PJ}^c}^2 + 4q_{PJ}^c N_{ideal}}\right)$$

In which p_{PJ}^c is the probability of filaments at exit i in the network which didn't make any wrong turns, and it can be calculated as $p_{PJ}^c = \left(1 - p_{PJ}\right)^{N_{PJ}}$, where N_{PJ} is the number of pass junctions along the exploration path; $q_{PJ}^c = 1 - p_{PJ}^c$; the other parameters are the same as the ideal scenario.

Estimating the confidence level for microtubules

Unfortunately, Konopik et al, [11] did not take into account the landing errors we observed for microtubules. Therefore, we use a method commonly used for probabilistic algorithms of the bounded-error probabilistic polynomial-time class[12]. Instead of analyzing the errors in the computation, one can repeat the same experiment multiple times to increase the reliability of the computation:

$$p_r = \alpha^m$$

where α is the confidence level of the individual experiment and m is the number of repetitions. Solving for m gives:

$$m = \frac{\log(p_r)}{\log(\alpha)}$$

Thus, an experiment with N agents and a significant result of confidence level α must be repeated m times, requiring

$$N_{required} = N * m = N * \frac{\log(1-p)}{\log(1-\alpha)}$$

agents.

Comparison to electronic computers

Let us assume that DRAM, as the most error prone part of electronic computers, dominates the overall error probability. The DRAM error rate in a large datacenter was measured to range from 25,000 to 70,000 errors per billion device hours per Mbit[13]. From this, we can estimate a confidence level for solving a computation with the same error probability as an electronic computer. For simplicity, we assume 1Mbit of memory and 1 second to complete the computation. Therefore, the error probability of the electronic computer is:

$$p_{DRAM} = \frac{70000}{1000000000 * 3600} = 2 * 10^{-8}$$

Which corresponds to the confidence level of 99.999998% that the computation is correct. Note, that this assumes a consumer-grade electronic

computer that does not use error correction code (ECC) DRAM.

Number of actin filaments needed to match the confidence of an electronic computer

Based on the approach described above, we can estimate the number of agents needed to match the confidence level of an electronic computer for our E32 NBC instances: $N_{actin,E32} = 1987$.

Number of microtubules needed to match the confidence of an electronic computer

In the presented work, we have demonstrated the instance E1024 is solved by the microtubule system at a confidence level of 95%. Therefore, to match the DRAM error probability, we need to repeat the experiment:

$$m_{E1024} \geq \frac{\log(2 * 10^{-8})}{\log(0.05)} \cong 6$$

times. Thus, we need $N_{microtubules,E1024} = 1554 * 6 = 9324$ microtubules to match the confidence level of an electronic computer.

S3 Estimated energy consumption per operation

Nicolau et al. estimated the energy consumption for their Subset Sum network to be 2 to $5 \cdot 10^{-14}$ J/operation, several orders of magnitude lower than the 2 to $6 \cdot 10^{-10}$ J/operation for an electronic computer[14]. In the following, we will use the same assumptions for the motor proteins: step size ~10 nm, 10^{-19} J/ATP molecule hydrolyzed, ~5 motors simultaneously propelling a 1 μm long filament. For the networks used in this manuscript, each network block, representing one subset encodes one operation. The average length of each block is ~8 junctions. With a junction size of ~10 μm, the average path that a filament needed to travel per operation is ~80 000 nm. Thus, we estimate the energy consumption per operation for our networks to be approximately $5 \cdot \frac{80\,000}{10} \cdot 10^{-19} = 4 \cdot 10^{-15}$ J/operation. Again, this is orders of magnitude less than an electronic computer. Note, that we did not take into account the energy required to polymerize the filaments, because they are re-useable (just like the CPU of an electronic computer is re-useable). Nor did we account for the energy required to manufacture the networks or the electronic computers, because (i) the networks are re-useable[15], (ii) can – in principle – be reprogrammed[16] and (iii) we do not think it is meaningful to compare the energy required for manufacturing a prototype to that of a mass-product.

Supplementary References

(1) Sundberg, M.; Rosengren, J. P.; Bunk, R.; Lindahl, J.; Nicholls, I. A.; Tågerud, S.; Omling, P.; Montelius, L.; Månsson, A. Silanized Surfaces for in Vitro Studies of Actomyosin Function and Nanotechnology Applications. *Analytical Biochemistry* **2003**, *323* (1), 127–138. https://doi.org/10.1016/j.ab.2003.07.022.

(2) Sundberg, M.; Balaz, M.; Bunk, R.; Rosengren-Holmberg, J. P.; Montelius, L.; Nicholls, I. A.; Omling, P.; Tågerud, S.; Månsson, A. Selective Spatial Localization of Actomyosin Motor Function by Chemical Surface Patterning. *Langmuir* **2006**, *22* (17), 7302–7312. https://doi.org/10.1021/la060365i.

(3) Salhotra, A.; Zhu, J.; Surendiran, P.; Meinecke, C. R.; Lyttleton, R.; Ušaj, M.; Lindberg, F. W.; Norrby, M.; Linke, H.; Månsson, A. Prolonged Function and Optimization of Actomyosin Motility for Upscaled Network-Based Biocomputation. *New J. Phys.* **2021**, *23* (8), 085005. https://doi.org/10.1088/1367-2630/ac1809.

(4) Clemmens, J.; Hess, H.; Lipscomb, R.; Hanein, Y.; Böhringer, K. F.; Matzke, C. M.; Bachand, G. D.; Bunker, B. C.; Vogel, V. Mechanisms of Microtubule Guiding on Microfabricated Kinesin-Coated Surfaces: Chemical and Topographic Surface Patterns. *Langmuir* **2003**, *19* (26), 10967–10974. https://doi.org/10.1021/la035519y.

(5) Ishigure, Y.; Nitta, T. Understanding the Guiding of Kinesin/Microtubule-Based Microtransporters in Microfabricated Tracks. *Langmuir* **2014**, *30* (40), 12089–12096. https://doi.org/10.1021/la5021884.

(6) van den Heuvel, M. G. L.; Butcher, C. T.; Smeets, R. M. M.; Diez, S.; Dekker, C. High Rectifying Efficiencies of Microtubule Motility on Kinesin-Coated Gold Nanostructures. *Nano Letters* **2005**, *5* (6), 1117–1122. https://doi.org/10.1021/nl0506554.

(7) Nitzsche, B.; Bormuth, V.; Bräuer, C.; Howard, J.; Ionov, L.; Kerssemakers, J.; Korten, T.; Leduc, C.; Ruhnow, F.; Diez, S. Studying Kinesin Motors by Optical 3D-Nanometry in Gliding Motility Assays. In *Microtubules, in vitro*; Academic Press, 2010; Vol. Volume 95, pp 247–271.

(8) Korten, T.; Chaudhuri, S.; Tavkin, E.; Braun, M.; Diez, S. Kinesin-1 Expressed in Insect Cells Improves Microtubule in Vitro Gliding Performance, Long-Term Stability and Guiding Efficiency in Nanostructures. *IEEE Transactions on NanoBioscience* **2016**, *15* (1), 62–69. https://doi.org/10.1109/TNB.2016.2520832.

(9) Castoldi, M.; Popov, A. V. Purification of Brain Tubulin through Two Cycles of Polymerization–Depolymerization in a High-Molarity Buffer. *Protein Expression and Purification* **2003**, *32* (1), 83–88. https://doi.org/10.1016/S1046-5928(03)00218-3.

(10) Schneider, C. A.; Rasband, W. S.; Eliceiri, K. W. NIH Image to ImageJ: 25 Years of Image Analysis. *Nat Methods* **2012**, *9* (7), 671–675. https://doi.org/10.1038/nmeth.2089.

(11) Konopik, M.; Korten, T.; Linke, H.; Lutz, E. Solving the Subset Sum Problem with a Nonideal Biological Computer. *New J. Phys.* **2021**, *23* (9), 095007. https://doi.org/10.1088/1367-2630/ac2005.

(12) Sipser, M. Introduction to the Theory of Computation. *ACM Sigact News* **1996**, *27* (1), 27–29.

(13) Schroeder, B.; Pinheiro, E.; Weber, W.-D. DRAM Errors in the Wild: A Large-Scale Field Study. *SIGMETRICS Perform. Eval. Rev.* **2009**, *37* (1), 193–204. https://doi.org/10.1145/2492101.1555372.

(14) Nicolau, D. V. Jr.; Lard, M.; Korten, T.; Delft, F. C. M. J. M. van; Persson, M.; Bengtsson, E.; Månsson, A.; Diez, S.; Linke, H.; Nicolau, D. V. Parallel Computation with Molecular-Motor-Propelled Agents in Nanofabricated Networks. *PNAS* **2016**, *113* (10), 2591–2596. https://doi.org/10.1073/pnas.1510825113.

(15) Rahman, M. A.; Reuther, C.; Lindberg, F. W.; Mengoni, M.; Salhotra, A.; Heldt, G.; Linke, H.; Diez, S.; Månsson, A. Regeneration of Assembled, Molecular-Motor-Based Bionanodevices. *Nano Lett.* **2019**, *19* (10), 7155–7163. https://doi.org/10.1021/acs.nanolett.9b02738.

(16) Schroeder, V.; Korten, T.; Linke, H.; Diez, S.; Maximov, I. Dynamic Guiding of Motor-Driven Microtubules on Electrically Heated, Smart Polymer Tracks. *Nano Lett* **2013**, *13* (7), 3434–3438. https://doi.org/10.1021/nl402004s.
